# Supplementary material for: Increasing amyloplast size in wheat endosperm through mutation of PARC6 affects starch granule morphology
Source: New Phytol. 2023 Jul 10;240(1):224–41. doi: 10.1111/nph.19118 (PMC10952435; doi:10.1111/nph.19118)
Supplement: Supplementary file 1 — Fig. S1 Molecular phylogenetic analysis of ARC6 and PARC6 gene families. Fig. S2 TaARC6 gene models and chloroplast morphology of the Ttarc6 mutant. Fig. S3 Growth and seed phenotype of Ttparc6 single homeolog mutants. Fig. S4 Photosynthesis parameters of Ttparc6 mutant plants. Fig. S5 Size distribution of purified starch granules from mature grains of the Ttparc6 single mutants. Fig. S6 Plant growth, grain morphology and starch phenotypes of the Ttparc6 mutant expressing the cTPmCherry amyloplast marker. Fig. S7 Analysis of properties of TtPARC6‐deficient Triticum turgidum starch. Fig. S8 Size distribution of starch granules of the Ttarc6 mutant and TtARC6 and TtPARC6 expression patterns during endosperm development. Fig. S9 Molecular phylogenetic analysis of PDV1 and PDV2 gene families and ARC6 mutant protein alignment. Methods S1 Phylogenetic analysis and gene models. Methods S2 Cloning and construct assembly. Methods S3 Transient transformation of Nicotiana benthamiana. Methods S4 Gas exchange. Methods S5 Starch purification, scanning electron microscopy and polarised light microscopy. Methods S6 Total starch quantification, starch composition and amylopectin structure. Methods S7 Analysis of chloroplast morphology. Methods S8 Microscopic analysis of amyloplast morphology in developing grain. Table S1 KASP‐markers for Ttparc6 and Ttarc6 genotyping. Table S2 Codon optimised DNA sequences of TaPARC1‐A1, TaARC6‐A1, TaPDV1‐1‐A1, TaPDV1‐2‐A1 and TaPDV2‐A1. Please note: Wiley is not responsible for the content or functionality of any Supporting Information supplied by the authors. Any queries (other than missing material) should be directed to the New Phytologist Central Office. [file NPH-240-224-s001.pdf]

***New Phytologist* Supporting Information**

Article title: Increasing amyloplast size in wheat endosperm through mutation of PARC6 affects starch granule morphology
Authors: Lara Esch, Qi Yang Ngai, J. Elaine Barclay, Rose McNelly, Sadiye Hayta, Mark A. Smedley, Alison M. Smith and David Seung
Article acceptance date: 08 June 2023

The following Supporting Information is available for this article:

Figure S1: Molecular phylogenetic analysis of *ARC6* and *PARC6* gene families.
Figure S2: *TaARC6* gene models and chloroplast morphology of the *Ttarc6* mutant.
Figure S3: Growth and seed phenotype of *Ttparc6* single homeolog mutants.
Figure S4: Photosynthesis parameters of *Ttparc6* mutant plants.
Figure S5: Size distribution of purified starch granules from mature grains of the *Ttparc6* single mutants.
Figure S6: Plant growth, grain morphology and starch phenotypes of the *Ttparc6* mutant expressing the cTPmCherry amyloplast marker.
Figure S7: Analysis of properties of *Tt*PARC6-deficient *Triticum turgidum* starch.
Figure S8: Size distribution of starch granules of the *Ttarc6* mutant and *TtARC6* and *TtPARC6* expression patterns during endosperm development.
Figure S9: Molecular phylogenetic analysis of *PDV1* and *PDV2* gene families and *ARC6* mutant protein alignment.
Table S1: KASP-markers for *Ttparc6* and *Ttarc6* genotyping.
Table S2: Codon optimized DNA sequences of *Ta*PARC1-A1, TaARC6-A1, *Ta*PDV1-1-A1, *Ta*PDV1-2-A1 and *Ta*PDV2-A1.
Method S1: Phylogenetic analysis and gene models
Method S2: Cloning and construct assembly
Method S3: Transient transformation of *Nicotiana benthamiana*
Method S4: Gas exchange
Method S5: Starch purification, scanning electron microscopy and polarised light microscopy.
Method S6: Total starch quantification, starch composition and amylopectin structure
Method S7: Analysis of chloroplast morphology
Method S8: Microscopic analysis of amyloplast morphology in developing grain

Figure S1: Molecular phylogenetic analysis of *ARC6* and *PARC6* gene families.
The tree with the highest log likelihood (-36614.75) is shown. The percentage of trees out of 1000 bootstraps in which the associated taxa clustered together is shown next to the branches. The tree is drawn to scale, with branch lengths and scale bar representing the number of substitutions per site. Full experimental procedures can be found in Methods S1.

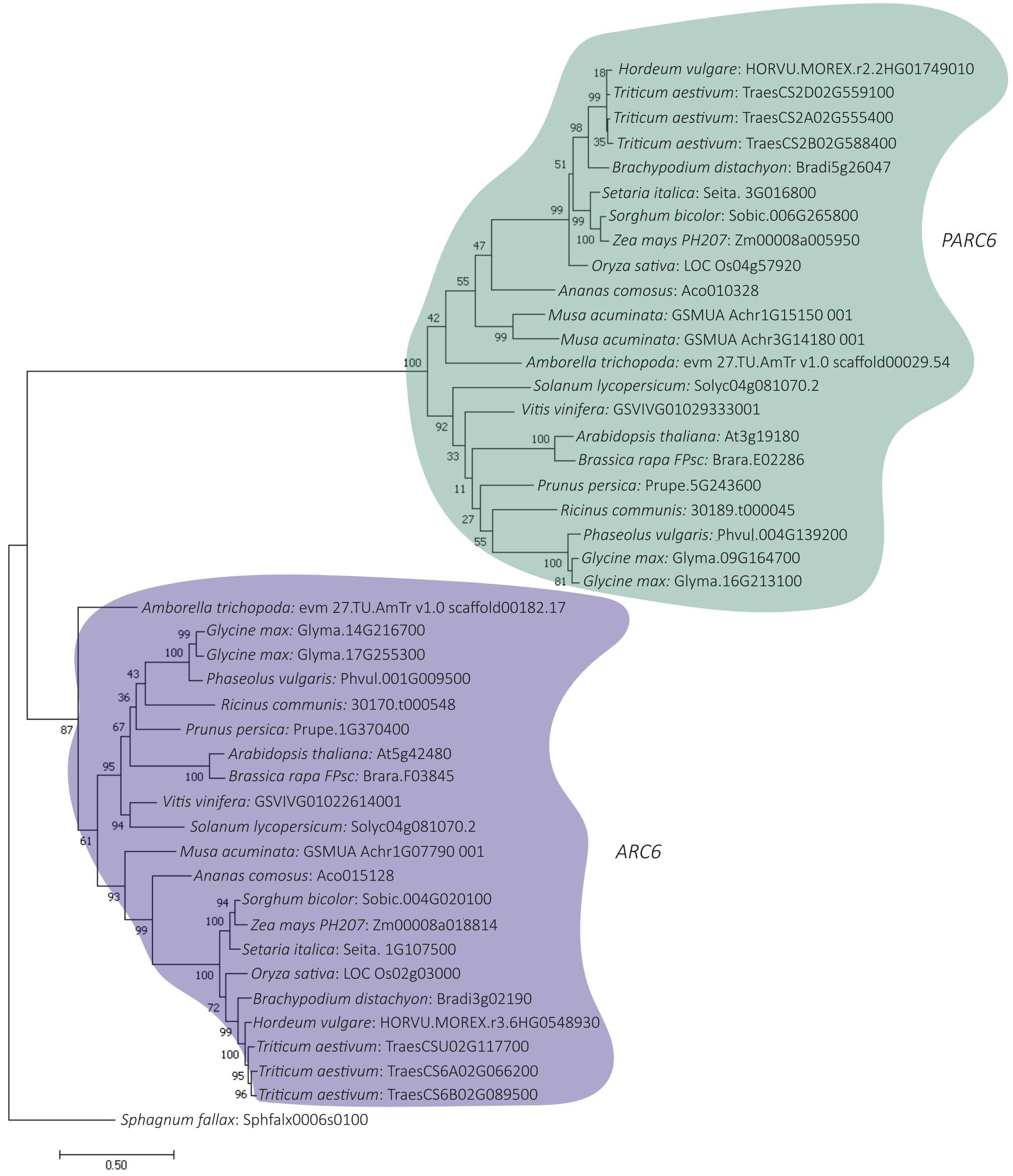

Figure S2: Wheat *TaARC6* gene models and chloroplast morphology of the *Ttarc6* durum wheat mutant.

(a) Schematic illustration of the gene models for the canonical transcripts of *TaARC6-A1*, *-B1* and *-D1* in bread wheat. Exons are represented as purple boxes and UTRs are represented as white boxes. Mutation sites in K3404 and K2205 are indicated by black lines and the resulting amino acid to stop codon (*) substitutions are annotated. Regions encoding domains are indicated by black horizontal lines (TM: Transmembrane, IMS: Inter Membrane Space).

(b-c) Images of mesophyll-cell chloroplasts in the third leaf of *Ttarc6* mutants seedlings. Images were acquired using confocal microscopy and are Z-projections of image stacks. Chlorophyll auto-fluorescence of the chloroplasts is shown in cyan. Bars = 10 µm.

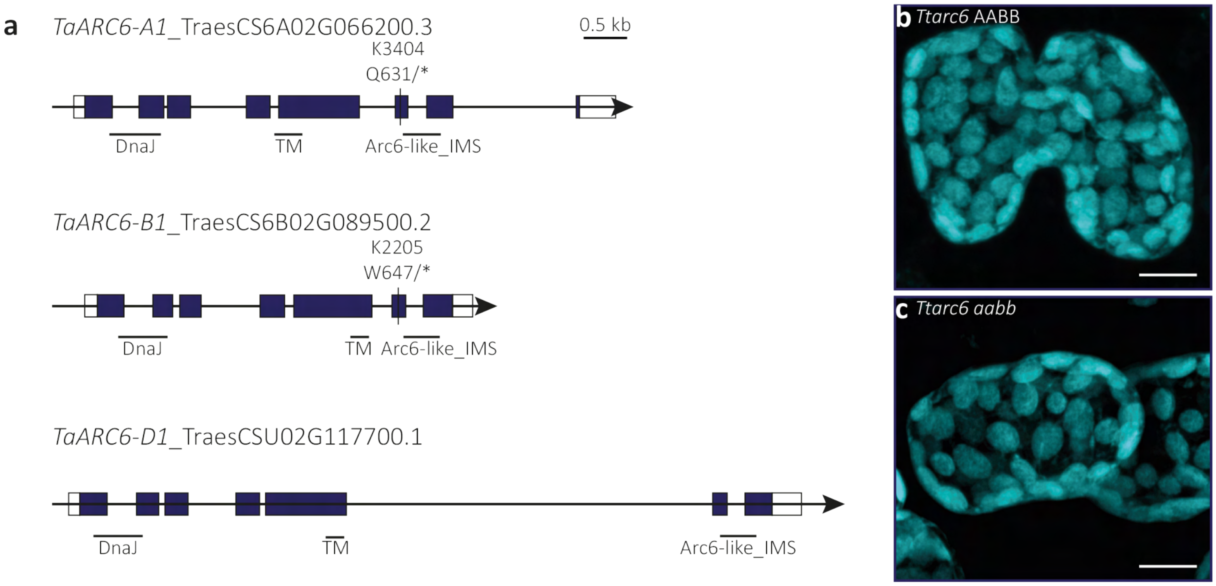

Figure S3: Growth and seed phenotype of *Ttparc6* single homeolog mutants of durum wheat.
(a, b) Photographs of 8-week-old *Ttparc6* double and single mutants (*Ttparc6-1 aabb*, *Ttparc6-1 aa*BB and *Ttparc6-1* AA*bb*) and the corresponding negative segregant (*Ttparc6-1* AABB); and *Ttparc6* backcrossed double and single mutant (*Ttparc6* BC *aabb*, *Ttparc6* BC *aa*BB and *Ttparc6* BC AA*bb*) and the corresponding negative segregant (*Ttparc6* BC AABB) and WT wheat (cv Kronos) plants. Bars = 10 cm.
(c-j) Images of mesophyll-cell chloroplasts in the third leaf of *Ttarc6* mutant seedlings. Images were acquired using confocal microscopy. Chlorophyll auto-fluorescence in the chloroplasts is shown in cyan. Bars = 10 µm.
(k) Photograph of 10 representative mature grains per genotype. Bar = 1 cm.
(l) The number of tillers per plant (Tiller no.) of mature plants (n = 6 - 19 per genotype). Significant differences between the lines as determined by Kurskal-Wallis One Way ANOVA on the Ranks all pairwise multiple comparison (Dunn's Method) ($p \leq 0.009$) are represented by different letters.
(m) Total grain weight harvested per plant (in g). Dots represent the total grain weight of individual plants (n = 6-19). Significant differences under a one-way ANOVA and all pairwise multiple comparison procedures (Tukey's test) are indicated with different letters ($P \leq 0.001$).
(n) Thousand grain weight (TGW) (in g). Dots represent calculated TGW of individual plants (*n* = 7-19) per genotype. Significant differences under a one-way ANOVA and all pairwise multiple comparison procedures (Tukey's test) are represented by different letters ($p \leq 0.001$).
(o) Grain size measured as seed area (in mm^2). Dots represent measurements for seeds of 3-19 plants per genotype. Significant differences under one-way ANOVA and all pairwise multiple comparison procedures (Tukey's test) are represented by different letters ($p \leq 0.05$).
(p) Total starch content as % (w/w). 3 technical replicates of 2 biological replicates per genotype. Significant differences under a one-way ANOVA and all pairwise multiple comparison procedures (Tukey's test) are represented by different letters ($p \leq 0.05$).
(q) Amylose content [% of total starch]. Dots represent 3 technical replicates of 3 biological replicates. Significant differences between the lines as determined by a one-way ANOVA on the ranks and all pairwise multiple comparison (Tukey's test) are represented by different letters ($p \leq 0.001$).
For all boxplots, the bottom and top of the box represent the lower and upper quartiles respectively, and the band inside the box represents the median. The ends of the whiskers represent values within 1.5x of the interquartile range, whereas values outside are outliers.

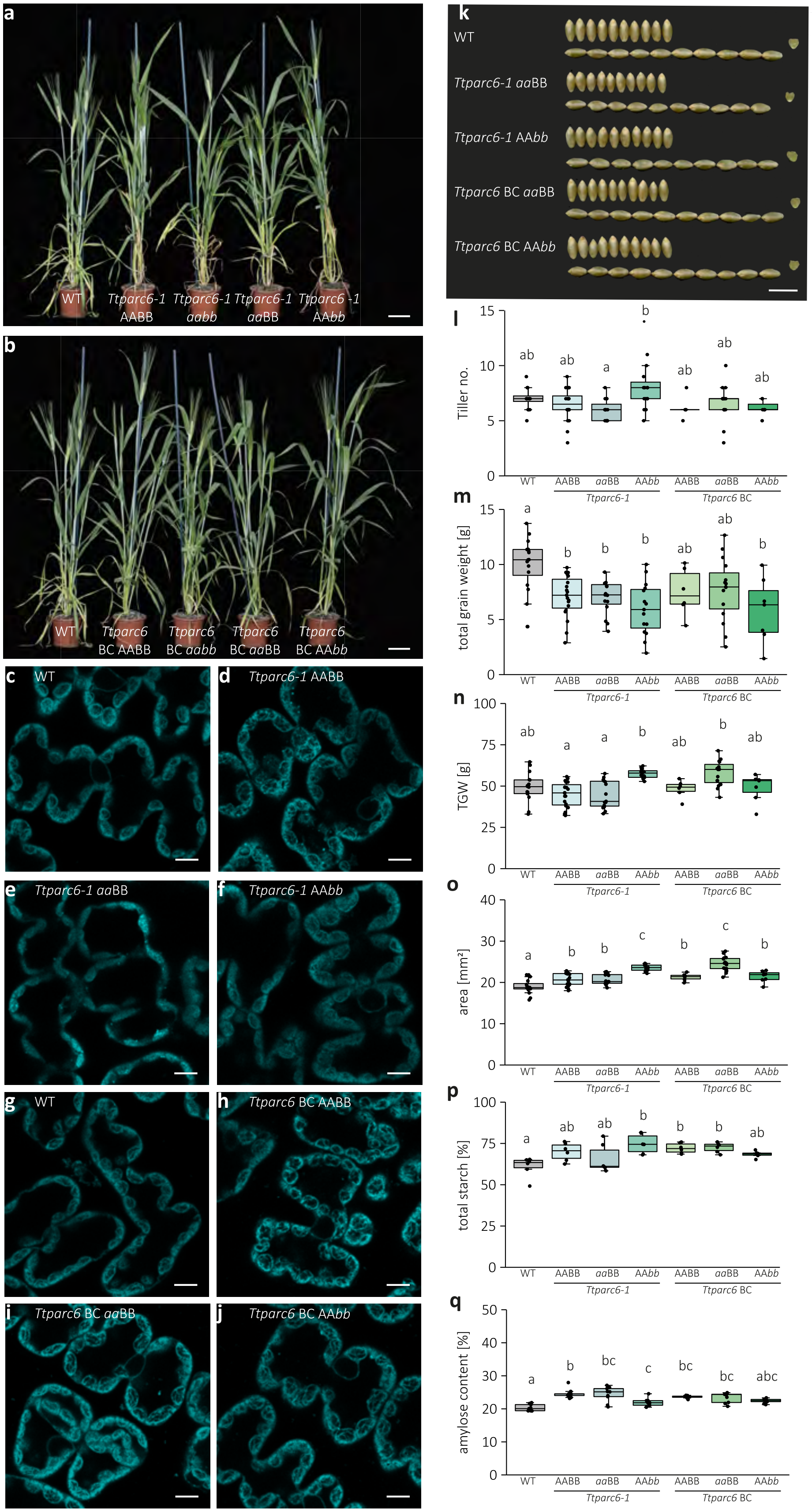
a
WT
Ttparc6-1 AABB
Ttparc6-1 aabb
Ttparc6-1 aaBB
Ttparc6 -1 AAbb
b
WT
Ttparc6 BC AABB
Ttparc6 BC aabb
Ttparc6 BC aaBB
Ttparc6 BC AAbb
c WT
d Ttparc6-1 AABB
e Ttparc6-1 aaBB
f Ttparc6-1 AAbb
g WT
h Ttparc6 BC AABB
i Ttparc6 BC aaBB
j Ttparc6 BC AAbb
k
WT
Ttparc6-1 aaBB
Ttparc6-1 AAbb
Ttparc6 BC aaBB
Ttparc6 BC AAbb
l
Tiller no.
m
total grain weight [g]
n
TGW [g]
o
area [mm²]
p
total starch [%]
q
amylose content [%]
WT AABB aaBB AAbb AABB aaBB AAbb
Ttparc6-1
Ttparc6 BC

Figure S4: Photosynthesis parameters of *Ttparc6* durum wheat mutants.

Light response and A/Ci curves were measured on the flag leaf of 40-46 day old plants in three separate plants (n = 3). Experimental procedures are in Methods S4.

(a-e) Light response curves were measured at ambient CO_2 levels (412 µmol m^{-2} s^{-1}). Lines represent the average and ribbons the standard error. The dotted line at A = 30 µmol m^{-2} s^{-1} is provided to aid comparison between genotypes.

(f) Assimilation rate at ambient light (280 µmol m^{-2} s^{-1}) and high light (2000 µmol m^{-2} s^{-1}).

(g) Estimation of Vcmax was extracted from the A/Ci curves using plant ecophys.package (R).

(h) Estimation of Jmax was extracted from the A/Ci curves using plant ecophys.package (R).

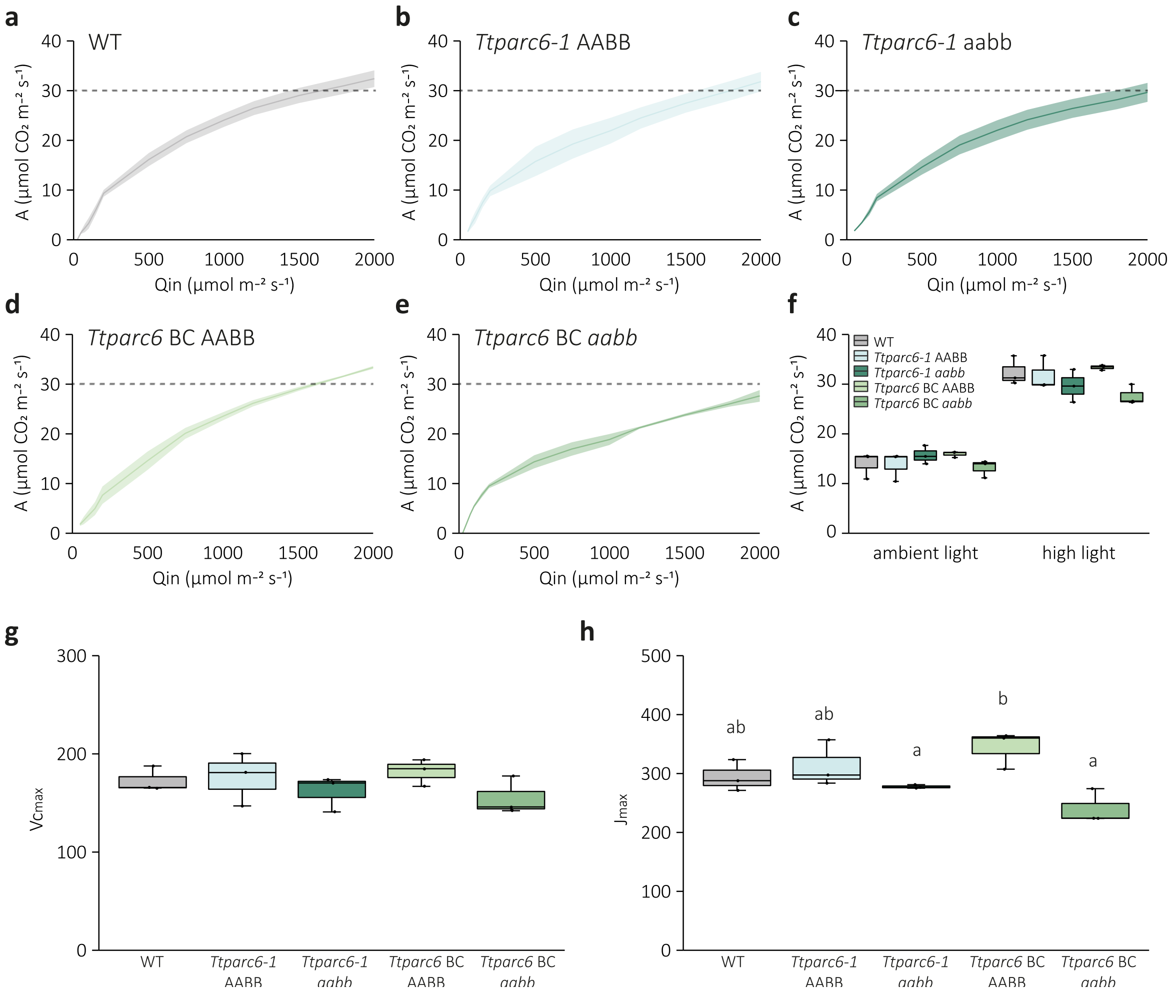

Figure S5: Size distribution of purified starch granules from mature grains of the durum wheat *Ttparc6* single mutants.

(a-b) Size distribution plots from Coulter counter analysis. The volume of granules at each diameter relative to the total granule volume was quantified using a Coulter counter. Values represent mean (solid line) ± SEM (shading) of three replicates using grains harvested from separate plants.

(c-h) Granule size parameters obtained from fitting a log-normal distribution to the B-type granule peak and a normal distribution to the A-type granule peak in the granule size distribution data presented in (a – b). Three biological replicates were analysed: **(c, d)** A-type granule diameter (in µm). Significant differences under a one-way ANOVA and all pairwise multiple comparison procedures (Tukey's test) are indicated with different letters ($p \leq 0.05$). **(e, f)** B-type granule diameter (in µm). Significant differences under a Kruskal-Wallis one-way ANOVA on ranks and all pairwise multiple comparison procedures (Tukey's test) are indicated with different letters ($p \leq 0.022$) for *Ttparc6-1* lines. Significant differences under one-way ANOVA and all pairwise multiple comparison procedures (Tukey's test) are indicated with different letters ($p \leq 0.05$) for *Ttparc6* BC lines. **(g, h)** B-type granule content by percentage volume. Significant differences under a one-way ANOVA and all pairwise multiple comparison procedures (Tukey's test) are represented with different letters ($p \leq 0.05$).

(i-m) Scanning Electron Microscopy of purified starch granules. Bars = 10 µm.

(n-r) Polarised light microscopy of purified starch granules. Bars = 10 µm.

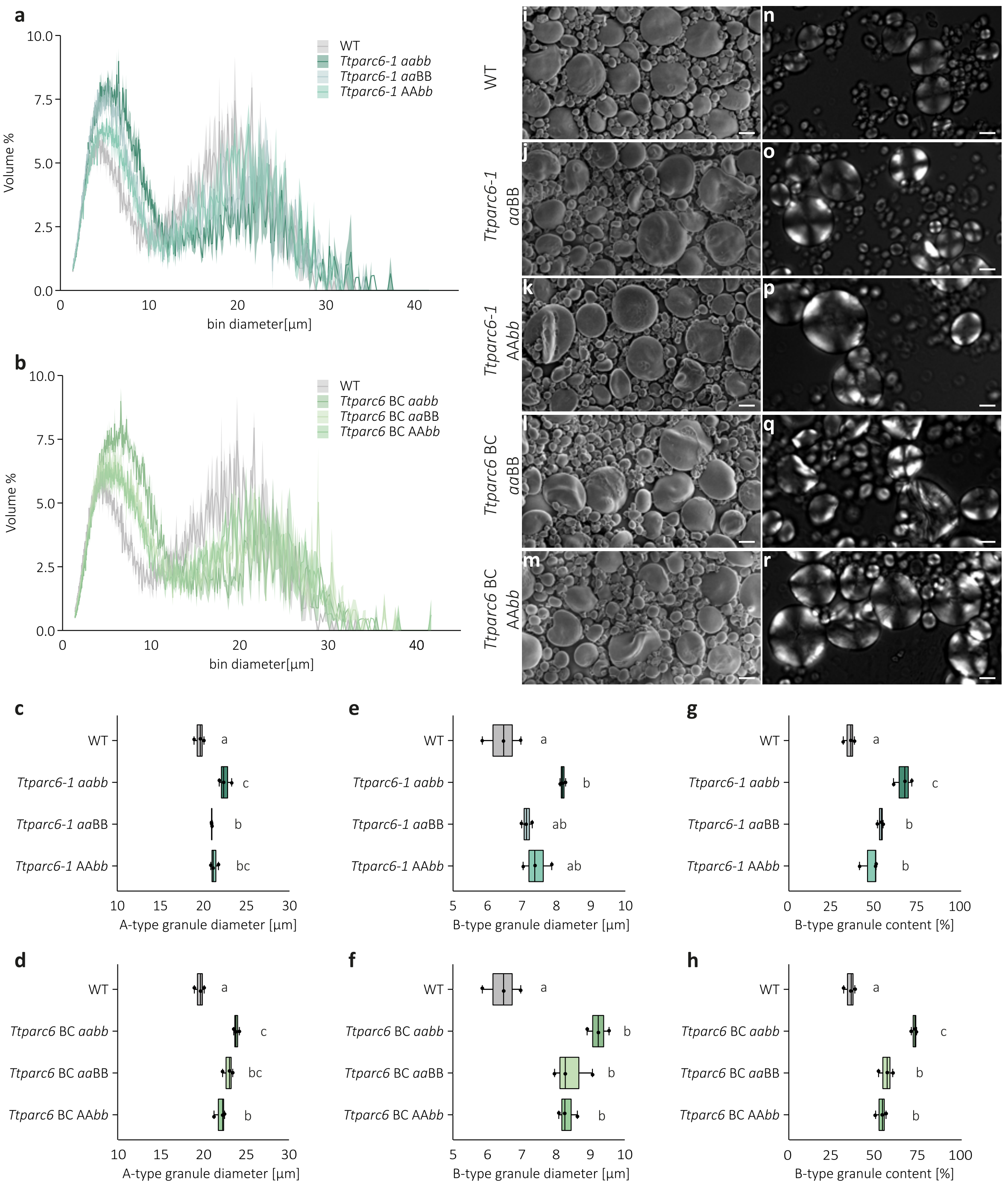
a
b
c
d
e
f
g
h
i
j
k
l
m
n
o
p
q
r
WT
Ttparc6-1 aabb
Ttparc6-1 aaBB
Ttparc6-1 AAbb
Ttparc6 BC aabb
Ttparc6 BC aaBB
Ttparc6 BC AAbb
Volume %
bin diameter[µm]
A-type granule diameter [µm]
B-type granule diameter [µm]
B-type granule content [%]
Ttparc6-1 aaBB
Ttparc6-1 AAbb
Ttparc6 BC aaBB
Ttparc6 BC AAbb

Figure S6: Plant growth, grain morphology and starch phenotypes of the *Ttparc6* durum wheat mutant expressing the cTPmCherry amyloplast marker.
(a) Photograph of 10 representative mature grains per genotype. Bar = 1 cm.
(b) Photograph of 8-week old transgenic cTPmCherry overexpressing mutant plants and corresponding negative segregants (*Taparc6-2 + cTPmCherry aabb* and *Taparc6-2 + cTPmCherry* AABB) and WT (cv Kronos) plants. Bar = 10 cm.
(c) The number of tillers per plant (Tiller no.) of mature plants (n = 10 – 16 per genotype). Significant differences between the lines as determined by Kurskal-Wallis One Way ANOVA on the Ranks all pairwise multiple comparison (Dunn's Method) ($p \leq 0.007$) are represented by different letters.
(d) Thousand grain weight (TGW) (in g). Dots represent calculated TGW of individual plants (n = 10 – 16 per genotype). There were no significant differences under a one-way ANOVA.
(e) Grain size measured as seed area (in mm^2). Dots represent measurements for seeds of 10-16 individual plants per genotype. Significant differences under a one-way ANOVA all pairwise multiple comparison procedures (Tukey's Test) are represented by different letters ($p \leq 0.05$).
(f) Total starch content as % (w/w). 3 technical replicates of 2 biological replicates per genotype. Significant differences under a one-way ANOVA and all pairwise multiple comparison procedures (Tukey's Test) are represented by different letters ($p \leq 0.002$).
(g) Amylose content [% of total starch]. Dots represent 3 technical replicates of 3 biological replicates. There were no significant differences between the lines determined by Kurskal-Wallis One Way ANOVA on the Ranks (p=0.317).
(h) Size distribution plots from Coulter counter analysis. The volume of granules at each diameter relative to the total granule volume was quantified using a Coulter counter. Values represent mean (solid line) ± SEM (shading) of three biological replicates.
(i-k) Scanning Electron Microscopy of purified starch granules from mature grain. Bars = 10 µm.
(l-n) Polarised light microscopy of purified starch granules from mature grain. Bars = 10 µm.
(o-q) Granule size parameters obtained from fitting a log-normal distribution to the B-type granule peak and a normal distribution to the A-type granule peak in the granule size distribution data presented in (h). Three biological replicates were analysed: **(o)** A-type granule diameter (in µm). Significant differences under a one-way ANOVA and all pairwise multiple comparison procedures (Tukey's test) are indicated with different letters ($p \leq 0.02$). **(p)** B-type granule diameter (in µm). Significant differences under a one-way ANOVA and all pairwise multiple comparison procedures (Tukey's test) are indicated with different letters ($p \leq 0.001$).
(q) B-type granule content by percentage volume. Significant differences under a one-way ANOVA and all pairwise multiple comparison procedures (Tukey's test) are represented with different letters ($p \leq 0.001$).
For all boxplots, the bottom and top of the box represent the lower and upper quartiles respectively, and the band inside the box represents the median. The ends of the whiskers represent values within 1.5x of the interquartile range, whereas values outside are outliers.

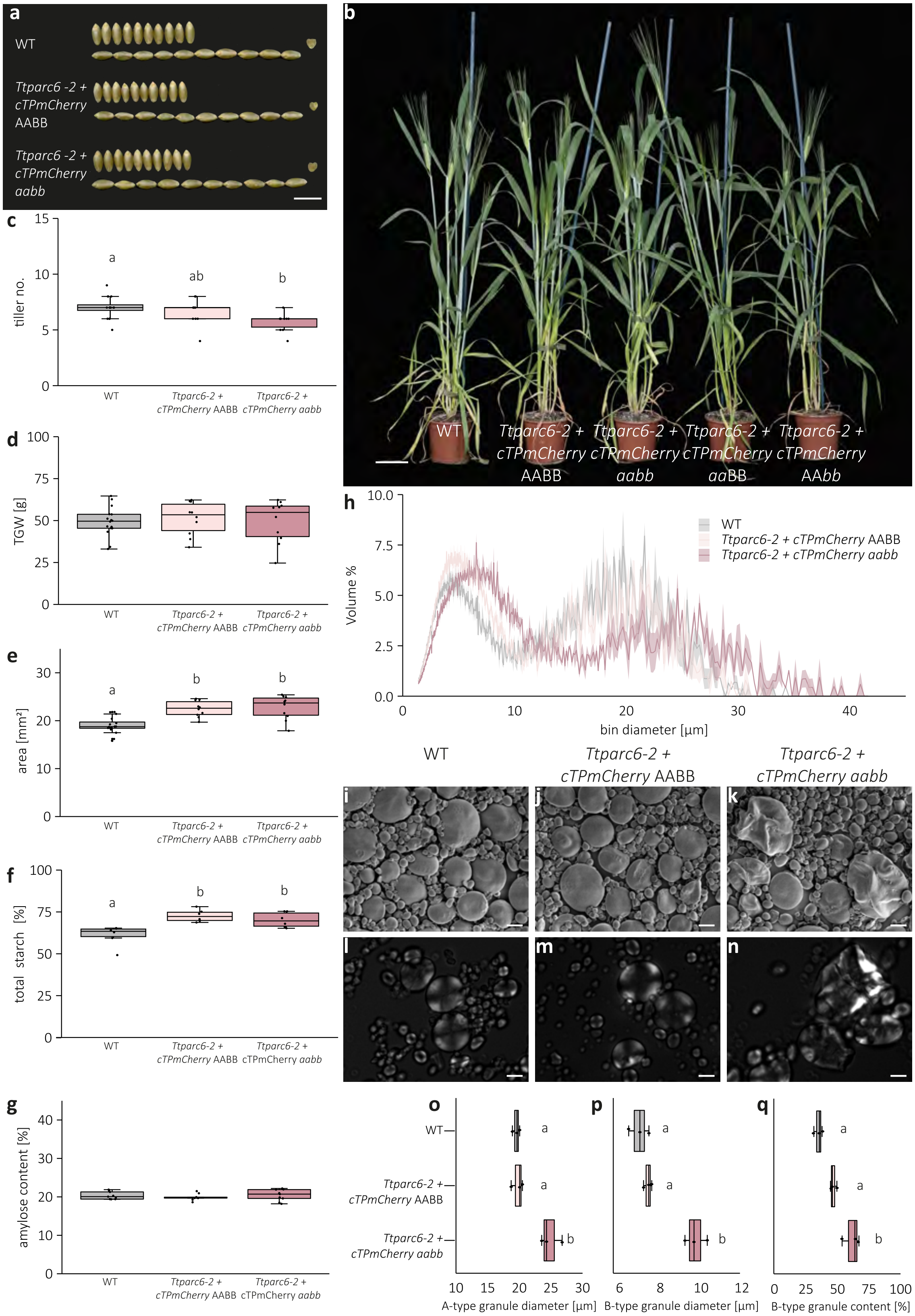

a
WT
Ttparc6 -2 + cTPmCherry AABB
Ttparc6 -2 + cTPmCherry aabb
b
WT
Ttparc6-2 + cTPmCherry AABB
Ttparc6-2 + cTPmCherry aabb
Ttparc6-2 + cTPmCherry aaBB
Ttparc6-2 + cTPmCherry AAbb
c
tiller no.
a
ab
b
d
TGW [g]
e
area [mm²]
a
b
b
f
total starch [%]
a
b
b
g
amylose content [%]
WT
Ttparc6-2 + cTPmCherry AABB
Ttparc6-2 + cTPmCherry aabb
h
Volume %
WT
Ttparc6-2 + cTPmCherry AABB
Ttparc6-2 + cTPmCherry aabb
bin diameter [µm]
WT
Ttparc6-2 + cTPmCherry AABB
Ttparc6-2 + cTPmCherry aabb
i
j
k
l
m
n
o
p
q
WT
Ttparc6-2 + cTPmCherry AABB
Ttparc6-2 + cTPmCherry aabb
a
a
b
A-type granule diameter [µm]
B-type granule diameter [µm]
B-type granule content [%]

Figure S7: Analysis of properties of *Tt*PARC6-deficient durum wheat starch.
(a) Amylose content [% of total starch] per genotype. Dots represent 3 technical replicates of 3 biological replicates. The bottom and top of the box represent the lower and upper quartiles respectively, and the band inside the box represents the median. The ends of the whiskers represent values within 1.5x of the interquartile range, whereas values outside are outliers. Significant differences between the lines as determined by Kurskal-Wallis One Way ANOVA on the Ranks all pairwise multiple comparison (Tukey's test) ($p \leq 0.001$) are indicated with different letters.
(b) Amylopectin chain length distributions. Starch was purified from grains and debranched with isoamylase prior to analysis using HPAEC-PAD. The y-axis represents the relative percentage of chains at each DP. Each line represents the average of three replicates per genotype (each using starch from grains harvested from a separate plant), and the shading represents the SEM.
(c-d) Rapid Visco Analyser (RVA) analysis of viscosity during gelatinisation. Analyses were conducted using: **(c)** Purified starch (1.5 g in 25 mL water). Values represent mean (solid line) ± SEM (shading) of three biological replicates, each using starch from grains harvested from a separate plant. The different genotypes produced viscographs that were generally similar, and there was no obvious difference in peak viscosity or the holding strength during cooling. Slight differences between genotypes were observed during cooling (retrogradation) and final viscosity, but these were not consistent for either *Ttparc6* double mutants or wild-type controls. **(d)** Whole flour (5 g in 25 mL water), where flour was produced by pooling a minimum of 3 biological replicates per genotype. The viscographs were more variable among genotypes, and there was no consistent effect that could be attributed to the mutant genotype.

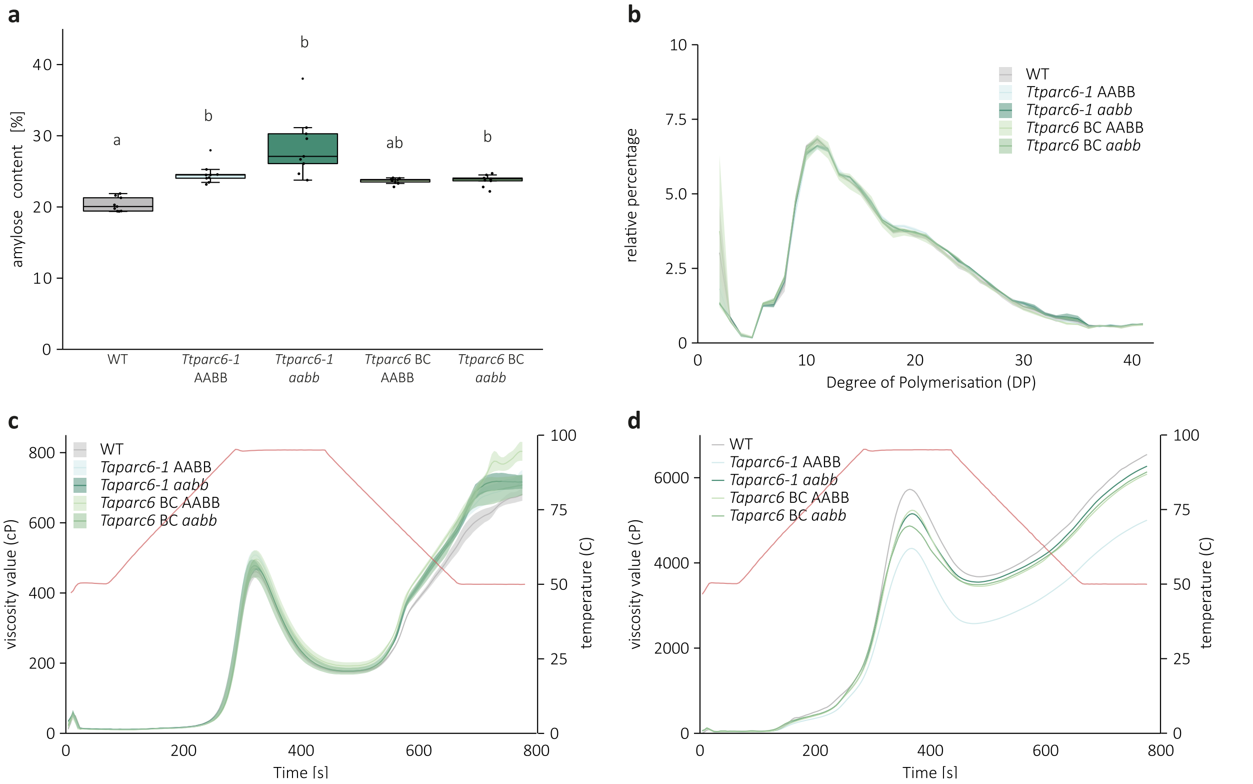

Figure S8: Size distribution of starch granules of the durum wheat *Ttarc6* mutant and *TtARC6* and *TtPARC6* expression patterns during endosperm development.

(a) Size distribution of purified starch granules from mature grain. The volume of granules at each diameter relative to the total granule volume was quantified using a Coulter counter. Values represent mean (solid line) ± SEM (shading) of three biological replicates.

(b-j) Average TPM values (Transcript per million) of the *PARC6, ARC6*, *PDV1-1*, *PDV1-2* and *PDV2* homeologs in the durum wheat endosperm. Values are the mean ± SEM of the n = 3 replicates. Normalised values were retrieved from Chen *et al.* (2022b).

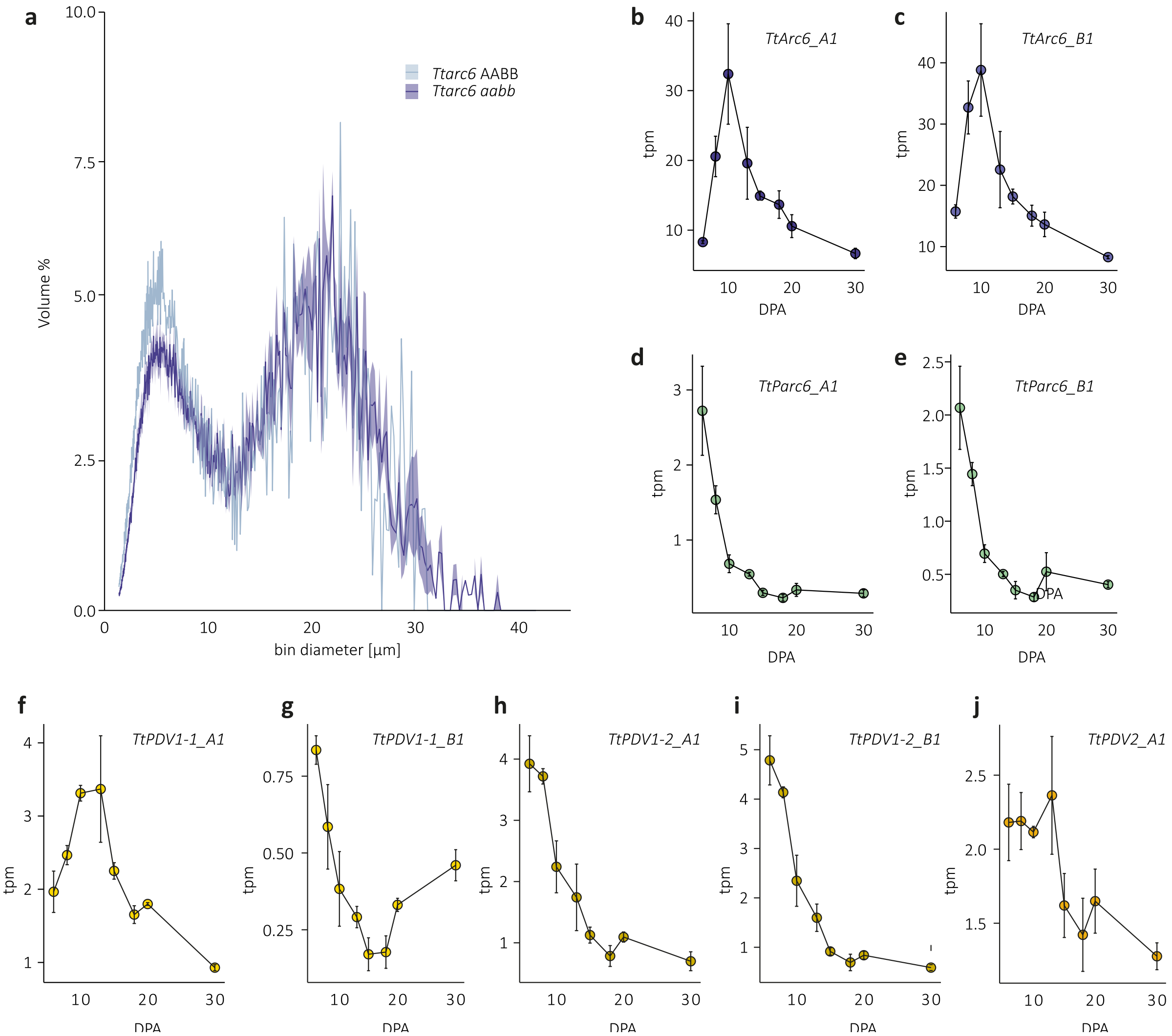

Figure S9. Molecular phylogenetic analysis of *PDV1* and *PDV2* gene families and *ARC6* mutant protein alignment.

(a) Schematic representation of multiple protein sequence alignment of wheat and Arabidopsis ARC6 protein sequences. Black lines indicate aligned sequence, gaps represent gaps in alignment. Degree of conservation is represented below, where red indicates high conservation and blue indicates low conservation. Brown arrows represent the region encoding the annotated transmembrane domain. Sequences and mutant sequences were retrieved from Ensembl plants and Glynn et al. (2008).

(b) Molecular phylogenetic tree of the PDV gene family was constructed from an amino acid alignment using the Maximum Likelihood method based on the JTT matrix-based model. The tree with the highest log likelihood (-20825.25) is shown. The percentage of trees out of 1000 bootstraps in which the associated taxa clustered together is shown next to the branches. The tree is drawn to scale, with branch lengths and scale bar representing the number of substitutions per site. Full experimental procedures can be found in Methods S1.

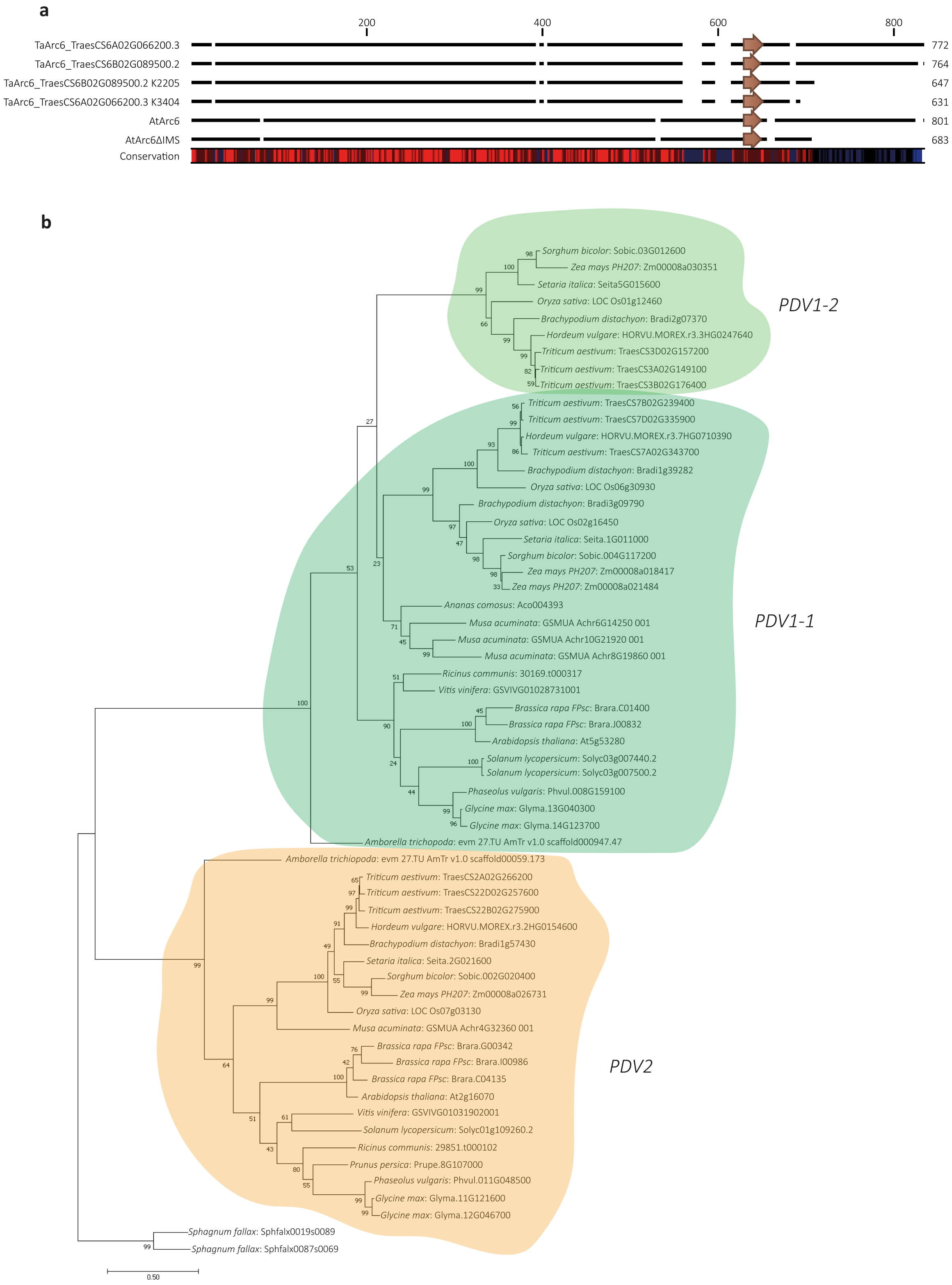

a
200
400
600
800
TaArc6_TraesCS6A02G066200.3
772
TaArc6_TraesCS6B02G089500.2
764
TaArc6_TraesCS6B02G089500.2 K2205
647
TaArc6_TraesCS6A02G066200.3 K3404
631
AtArc6
801
AtArc6ΔIMS
683
Conservation
b
PDV1-2
Sorghum bicolor: Sobic.03G012600
Zea mays PH207: Zm00008a030351
Setaria italica: Seita5G015600
Oryza sativa: LOC Os01g12460
Brachypodium distachyon: Bradi2g07370
Hordeum vulgare: HORVU.MOREX.r3.3HG0247640
Triticum aestivum: TraesCS3D02G157200
Triticum aestivum: TraesCS3A02G149100
Triticum aestivum: TraesCS3B02G176400
PDV1-1
Triticum aestivum: TraesCS7B02G239400
Triticum aestivum: TraesCS7D02G335900
Hordeum vulgare: HORVU.MOREX.r3.7HG0710390
Triticum aestivum: TraesCS7A02G343700
Brachypodium distachyon: Bradi1g39282
Oryza sativa: LOC Os06g30930
Brachypodium distachyon: Bradi3g09790
Oryza sativa: LOC Os02g16450
Setaria italica: Seita.1G011000
Sorghum bicolor: Sobic.004G117200
Zea mays PH207: Zm00008a018417
Zea mays PH207: Zm00008a021484
Ananas comosus: Aco004393
Musa acuminata: GSMUA Achr6G14250 001
Musa acuminata: GSMUA Achr10G21920 001
Musa acuminata: GSMUA Achr8G19860 001
Ricinus communis: 30169.t000317
Vitis vinifera: GSVIVG01028731001
Brassica rapa FPsc: Brara.C01400
Brassica rapa FPsc: Brara.J00832
Arabidopsis thaliana: At5g53280
Solanum lycopersicum: Solyc03g007440.2
Solanum lycopersicum: Solyc03g007500.2
Phaseolus vulgaris: Phvul.008G159100
Glycine max: Glyma.13G040300
Glycine max: Glyma.14G123700
Amborella trichopoda: evm 27.TU AmTr v1.0 scaffold000947.47
PDV2
Amborella trichiopoda: evm 27.TU AmTr v1.0 scaffold00059.173
Triticum aestivum: TraesCS2A02G266200
Triticum aestivum: TraesCS22D02G257600
Triticum aestivum: TraesCS22B02G275900
Hordeum vulgare: HORVU.MOREX.r3.2HG0154600
Brachypodium distachyon: Bradi1g57430
Setaria italica: Seita.2G021600
Sorghum bicolor: Sobic.002G020400
Zea mays PH207: Zm00008a026731
Oryza sativa: LOC Os07g03130
Musa acuminata: GSMUA Achr4G32360 001
Brassica rapa FPsc: Brara.G00342
Brassica rapa FPsc: Brara.I00986
Brassica rapa FPsc: Brara.C04135
Arabidopsis thaliana: At2g16070
Vitis vinifera: GSVIVG01031902001
Solanum lycopersicum: Solyc01g109260.2
Ricinus communis: 29851.t000102
Prunus persica: Prupe.8G107000
Phaseolus vulgaris: Phvul.011G048500
Glycine max: Glyma.11G121600
Glycine max: Glyma.12G046700
Sphagnum fallax: Sphfalx0019s0089
Sphagnum fallax: Sphfalx0087s0069
0.50

Table S1: KASP-markers for genotyping durum wheat *Ttparc6* and *Ttarc6* mutations.

Line	Specificity	VIC/HEX or FAM tail	Genome-specific sequence
Kronos1265	WT	gaaggtcggagtcaacggatt	cgagaagagtccttttgagctctc
	Mutant	gaaggtgaccaagttcatgct	cgagaagagtccttttgagctctt
	Common		gcctatccgttgatccctggc
Kronos2369	WT	gaaggtcggagtcaacggatt	tgcaacataccagtgactg
	Mutant	gaaggtgaccaagttcatgct	tgcaacataccagtgacta
	Common		cagcttcaaagtaatggaagttccaattcaaga
Kronos2205	WT	gaaggtcggagtcaacggatt	ccttggatttgatactctgc
	Mutant	gaaggtgaccaagttcatgct	ccttggatttgatactctgt
	Common		aatttgtttctgaaatcatagtgcc
Kronos3404	WT	gaaggtcggagtcaacggatt	gcatccattctaggaatctg
	Mutant	gaaggtgaccaagttcatgct	gcatccattctaggaatcta
	Common		aatttgtttctgaaatcatagtgct

WT: wild-type

Table S2: Codon optimized DNA sequences of wheat *Ta*PARC1-A1, TaARC6-A1, *Ta*PDV1-1-A1, *Ta*PDV1-2-A1 and *Ta*PDV2-A1.

codon optimized sequences
>TaParc6_A1_CDS_attL_MluI_codon_optimized_Nbenth<br>ACGCGTCAAATAATGATTTTATTTTGACTGATAGTGACCTGTTCGTTGCAACAAATTGATGAGCAATGCTTTTTTATAATGCC<br>AACTTTGTACAAAAAAGCAGGCTTCACCATGGCTATGCCTACTCCTGCTGCTGCGCTTCTTCATCCATCTTCTGCTGTGGTAG<br>CTGCTCCTTCTCCTTCTACGTCTTCTTCTGCTAGACGGTCTGCTCCTTCTTCTTCTTCTTCTTCTGCGAGAAGAGGTGGTAATG<br>CTTCTGCTGGTAGAGGTGCCGCTGTTAGACCTAGAGTTGCTGGTGCGGCTGCACCTGTGACAGCTGCTGCTGCTGCTGAAG<br>GTTGTGGTAGACAAGAGCCTCCTGCGGCCCCTGCTGTTGAAATTCCTGTTACTTGTTATCAAATTCTTGGTGTTACTGAAAA<br>GGCTGAAAAGGATGAAATTGTTAAGTCTGCTATTGAACTTAGAAAGTCTGAAATTGAAGATGGTTATACTGAAGAAGTTTC<br>TACTTGTAGACAAGCTCTACTACTTGATGTTAGAGATAAGCTTCTTTTGAACAAGAATATGCTGGTTCTACTAGAGCTAAG<br>GTTCCTCCTAGATCTTCTCTTCATATTCCTTGGTCTTGGCTTCCTGCTGCTCTTTGTGTTCTTCAAGAAGTTGGTGAAGAAAAG<br>CTTGTTCTTGATATTGGTCAAGCTGCTCTTAGAAGAACTGATTCTAAGCCTTATGCTCATGATGTTCTTCTTGCTATGGCTCTT<br>GCTGAATGTTCTATTGCTAAGGCTTCTTTTGAAAAGTCTAAGGTTTCTCTTGGTTTTGAAGCTCTTGCTAGAGCTCAATATCTT<br>CTTAGAAAAAAACCATCTCTTGAAAAGATGCCTCTTCTTGAACAAATTGAAGAATCTCTTGAAGAACTTGCTCCTGCTTGTAC<br>TCTTGAAGTTCTTTCTCTTCCTAGAACTCCTGAAAATTCTGAAAGAAGAAGAGGTGCTATTGCCGCTTTGTGTGAGCTGCTTG<br>GTCAAGGTCTAGATGTTGAATCTTCTTGTAGAGTTCATGATTGGCCTTACTTTTTAGGCCAAGCGATGGACAAATTGCTTGC<br>TACCGAAATTGTTGAACTTCTTTCTTGGGATTCTCTTGCTACTACTAGAAAGAATAAGAAGTCTCTTGAATCTCAATCTCAAA<br>GAGTTGTTGTTGATTTTAATTGTTTTTATAGAGCTATGCTTGCTCATCTTGCTTCTGGTTTTTCTACTAGACAAACTGAACTTA<br>TTTCTAAGGCTAAGACTATTTGTGAATGTCTTGTTGCTTCTGAAAATACTGATCTTAAGTTTGAAGAATCTTTTTGTTCTTTTC<br>TTCTTGGTGAAGAATCTGGTGCTACTGTTTTCGAAAAGCTTCAACAACTTCAATCTAATGGTTCTTCTAATTCTAGAAATTAT<br>GGTCTTGCTAAGAAGAAGGATTCTTCTGATAAGGTTACTGTTAATCAATCTCTTGAACTTTGGCTTAAGGAAGTTGCTCTTTC<br>TAGATTTGCTGATACTAGAGATTGCCCCCCTTCTCTTGTTAATTTTTTTGCTGCTCCTAAGAGACTTATTTCTACTTCTAAGCA<br>AAAGCTTGGTGCTACTAGAAGAGTTCTTCTTTCTTCTCAAACTCCTTCTTCTGCCTCTACGTGTAATAGAACTTCTGGTCAACA<br>AAATCCTAGACTTAATTCTACTTCTCATCTTGGTGAAGCTGTTAAGCAACTTGCTCCTACTACTCTTGGTGGTCAAGGTTCTA<br>CTGATAGACCTGTTAATGGTCTTTCTACTACTTCTGTTCCTCTTAAGAGAAATCCTGGTTCTCATCCTGTTAGAACTCTTGAAT<br>CTTGGGGTCTTACTGGTGATGTTATTGGTAAGATTGCTTATACTGCTGTTCTTGGTCTTGCTCTTTTTGGTACTCTTAAGCTTC<br>TTAGATTTCAATTTGGTAATACTAAGCCTGCTCCTTCTACTAGAGAATCTGCTGCTACTAGTTCTCTCAACGAAGCTTCTCCTT<br>CTGAAGGTTCTTTTATTTCTTCTAGAGTTAGAGAACAATTCGAAAAGCTTTCCAAAATGCTTTGGCTTAATAATAGAGTTCAT<br>CTTAGATCTGAAAGATCTGATCTTTCTCCTGGTTCTTCTGATGTTGCTGCTATTGCTAGAAAGGAAAGAATGTCTCTTCAAGA<br>AGCTGAAGCTCTAGTTAAGCAATGGCAAGATATTAAGTCGGAAGCTTTAGGTCCTGATTATGAAATTGATATGCTTTCTGAA<br>GTTCTTGATGGTTCTATGCTTTCTAAGTGGCAAGATCTTGCTCTTTCTGCTAAGGATCAATCTTGTTATTGGAGATTTGTTCTT<br>CTTAATCTTTCTGTTGTTAGAGCTGAAATTCTGCTTGATGAAGCCGGTGATGGTGAAGTTGCTGAAATTAATGCTGTTCTTG<br>AAGAAGCTGCTGAACTTGTTGATGATTCTCAACCTAAGAAGCCCTCTTACTATTCTACTTATGAAGTTCAATATTCTCTTAGA<br>AGACAAGATGATGGTTCTTGGAAGATTTGTGAAGCTGCTGTCAGAGACTTATCTGACCCAGCTTTCTTGTACAAAGTTGGCA<br>TTATAAGAAAGCATTGCTTATCAATTTGTTGCAACGAACAGGTCACTATCAGTCAAAATAAAATCATTATTTGCACGCGT
>TaArc6_A1_CDS_GW_codon_optimized_Nbenth<br>ggggacaagtttgtacaaaaaagcaggcttcATGGAGGGTCTCCACAACCTGCTTGCAAGACCTAATTCTGCACCTTTAGCATTTTCTC<br>CACCTAGACCAAGACCAAGAAGAAGGCCACCTGTTGCATGTCGAGCAGCTAGCCGCTGGGCCGACCGCCTCTTCGCCGACT<br>TCCATTTATTGCCTACAGCAGCAGCTCCTGAACCACCTGCAGCAGCTCCTGCTGGTGTATCTGCATCACCTTGTGTACCTCTA<br>TTTCCAGATGCCGCCGACCGCTCCCTTCCCCTCCAGGTCGACTTCTACAAGGTTCTCGGCGCGGAACCGCATTTCCTCAGCG<br>ACGGCGTCAGGCGGGCCTTCGAGGCGAGGGCGGCCAAGCCACCGCAGTACGGCTACAACACAGATACCCTTGTTGGCCGT<br>CGGCAAATACTGCAGCTTGCACATGATACTCTCACAAACCAGAGCTCCCGCACCGAGTATGACCGCGCGCTCTCTGAGGAC<br>CGTGGCATGGCGCTCACATTGGATGTTGCTTGGGACAAGGTTCCGGGTGTGCTGTGTGCCCTTCAGGAGGCTGGGGAGGC<br>ACAGGCAGTGCTCGCAATTGGAGAGCACTTGCTGCAGGACCGCCCGCCCAAGCAGTTCAAACAGGATGTGGTGCTGGCAA<br>TGGCTCTGGCCTATGTGGATCTATCAAGGGACGCAATGGCGGCTAGCCCACCAGACGTAATCCGCTGCTGTGAGGTGCTTG<br>AAAGGGCTCTCAAGCTCTTGCAGGAGGATGGGGCAATCAATCTCGCACCTGATCTGCTTTCACAAATTGATGAAACTCTGG<br>AGGAGATCACACCTCGTTGTGTTTTGGAGCTTCTTGCCCTTCCTCTTGATGAAAAGCACCAGAGTAAACGCCAAGAAGGTCT<br>TCGTGGTGTGAGAAACATTTTGTGGAGTGTTGGTAGAGGAGGTATTGCTACTGTTGGAGGAGGATTTTCGCGTGAAGCCT<br>ACATGAATGAGGCCTTTTTGCAGATGACATCAGCGGAGCAGATGGATTTCTTTTCAAAAACGCCAAATAGCATACCACCTG

AATGGTTTGAAATCTATAGTGTGGCACTCGCAAATGTTGCTCAAGCAATTGTAAGTAAAAGGCCAGAGCTCATCATGGTGG
CAGATGATCTTTTCGAACAGCTCCAGAAGTTCAATATAGGTTCTCAATATGCTTATGATAATGAATTGGATCTTGTGTTGGA
AAGGGCACTTTGCTCATTGCTTGTGGGAGACATTAGCAACTGCAGAATTTGGCTTGCGATTGATAATGAATCCTCACCACAT
AGAGACCCCAAAATTGTAGAGTTTATTGTGAACAACTCTAGCATTGACCACCAGGAGAATGATCTTCTTCCAGGCCTGTGTA
AGCTTTTGGAGACTTGGCTTGTCTCAGAGGTTTTCCCTAGGAGCAGAGATACTCGAGGCATGCAGTTTACACTTGGAGACT
ACTACGATGATCCACAAGTTTTAAGCTACCTAGAAATGATGGAAGGTGGTGGTGCTTCTCATTTGGCTGCTGCTGCTGCTAT
AGCAAAACTCGGTGCTCAAGCTACAGCTGCGCTTGGTACCGTGAAATCAAGTGCTATCCAAGCATTCAACAAGATTTTTCCA
TTGATAGAACAGCTAGATCGATCAGACATGGAGAATCCTAATGATGGCCCTGAGGAATCTGTCAATAAATTTGACCAGAAA
ACTATTATGGGATTTGATATCCGTGATTCCAAAAATGCTGCCCTGAAGATTGTCTCTGCCAGTGCATTATTTGCTCTGATGAC
AGTAATAGGCATGAAGTACTTGCCTCGTAACAAGGTGCTCCCTGCTATTAGAAGCGAGCATAAGTCCATGACAGTTGCTAA
TGTTGTTGACTCAGTTGATGATGATGCACCAGATGAGCCAATACAGATTCCTAGAATGGATGCGAATCTGGCAGAAGGTAT
TGTTCGCAAGTGGCAGAGTATCAAATCCAAGGCCTTGGGATCAGATCATTCTTTGGAATCATTGCAAGAGGTTCTTGATGG
CAACATGCTGAAGGTATGGAGGGACCGAGCAGCAGAGATCGAGCGCAAAGGCTGGTTCTGGGACTACACGCTGTCCGAC
GTGGCGATTGACAGCATCACCGTCTCCCTGGACGGACGACGGGCGACTGTGGAGGCGACAATTGAGGAGGCAGGCCAGC
TTACCGATGCAACCGACCCCAGGAACAACGATTTGTACGACACTAAGTACACCACCCGGTACGAGATGACCTTCACTGGAC
CAGGAGGGTGGAAGATAACAGAAGGTGCGGTCCTCAAGTCGTCAgacccagctttcttgtacaaagtggtcccc

>TaPDV1-1_A1_CDS_GW_codon_optimized_Nbenth
ggggacaagtttgtacaaaaaagcaggcttcaccATGACCGCTTCTTCACCACCACACGATACCAAACAGTTGGGGACACAAAGGGAG
AAGGAAAAGGGTAACCCAAAGAAGAAAGTTATATTGTCAACCGAAGGAGAAGAGAAGAAGAGCTCTACACGAAGGGCAC
CAGGTTGCCCTCGAGCTTGCTTACCTTCATCTGCCCCCCATCCCCCTTCCTCACTGCAGAGGAGAAGGGCCAACCGTGTTAA
TGAGATGCGTTGGGACTGGGAGACACCTGCAACAGAAGCAGAGGCAGAAGCTTTGCAGGAAAGGATATGGGATCTTCAT
GATAAGCTCTCTCATGCTATTCTCGCCCTGTCCGCCTGTGCAGGTTTACCGGCTTGCAGGTGCCGTGGCGCACCCAATGGCC
ACGTAATACTGAAGGGTCAAAGACCCCCCAAGGAGGTGGACACGTAGACTTGGCAGCTGCAGCTGCCGCCATGGCTGAC
GCCAGGGGATTGCACGCCATCAGGACCGCCCTGGAAGACCTCGAAGGACACCTGCACTTCCTTCGTGACGTTCAATCTCAA
CAACGAGCTGACCGTGATGCAGCAATAGCTAGAGTGCAGCAGAGCAGAATACTCCTTGCAGCAAGGTTAGCCGAACACAG
GGGCAAGGGCCATGGAGTGATCGAAGAGGCACTTGGATTTGTCGGTGATGTGCGTGATAAGTCACATTTTGTAAGTCCGG
AGGACGTCTATGGTATGCACTCCCAGAGTGGTGAGGACGAGGAAGATAGGCGTGGGCATGGTTCCAACATGGTGGTTCGA
GTGGTTTCCTGCTCCTTCGCACTCGCCAAAAATATCTTGAGGTTTGAGACCATGGGCAGCGTGCTGGGTAACGCCACTGTGT
TCGCTGTAAGTATGCTGACTTTCCTCCAACTCCACCAGTTAGCTTCAGGAAAGCAAATGCCCGCCGTACAATACCGTAGGAC
AGATAACGTTAGCTTATCCGGCGGCTCTAGAAAGGATACAAAAGGCAAACACCTCGAGGTGCTTCTCGCCAGGGGTgaccca
gctttcttgtacaaagtggtcccc

>TaPDV1-2_A1_CDS_GW_codon_optimized_Nbenth
ggggacaagtttgtacaaaaaagcaggcttcaccATGGAGCCGGAAGAAGCCGAAGCAGTCCTCGAAACAATCTGGGACTTGCACGA
CAAGGTTTCTGACGCCATTCATGCTCTGTCTAGGGCCCACTTTCTCAGAGCCGTGCGAAGAAGAGCAGGTGGCAAACCAGC
CGGTGTAGTGCACATAAAGGGGGTCCCTGCCGATGGCGACGAGGCCGCCGATCTTAACGCTGTAGCCGAAGAAGCTAGGA
GTTTACATGCCATCCGAGCAGCTTTGGAAGACTTGGAGGATCAGTTCGAGTGTTTCCTCGCTGTTTGTTCCCAACAACAGGC
TGAAAGGGATATTGCATTAGCCCGTTTGCAACAATCTCATATCATGTTAACAATACGTCTCAAAGAACACCATGGAAACAAT
CATAAGGTCATAGATGAAGCATTGGACTTCGTGCACAATGTGTATCATGACTTTTGGAGTTTCTTATCCGTGAATAAACCTG
AAAAAAGTAGGAGCCACTCAGGCGCAAACTCTACTAAGGAGACTGGGGACGGGAGCAATTTTTTAGGCTGGATGGTATCT
TCATCCTTGGATGCCGTTAGAAATTCATTTAACGTCAAAAACTTCGGCGGCTTCCTGGGCAACAGCGCCGTTTTCGCTGTCG
GGATGATTACTATGCTTCAGCTTCACCTGTTAAGTTCAGGTGAGCAGTCATCTTCATGTGGAAAATACAGCTACAGAAGAAT
CAATCGAGATGACAGTTCACAATCACTTGCTGGTCGTTCACGATCCTCCCATCTTGACGTGTTTCTTGCAAAGAGTgacccagc
tttcttgtacaaagtggtcccc

>TaPDV2_A1_CDS_GW_codon_optimized_Nbenth
ggggacaagtttgtacaaaaaagcaggcttcaccATGGAGGGTGAAGAGGAAATTGGGCTCGTCCTCGCCCGAGCCAGTGATCTCAG
GTCACGAATCTCTGCATGTGCAGCCGCAGCTCGACCTCCTCCAAGATTAGGAGCTGGAGAGGAAGACGATGGAGGCGAAG
AGGAAGAAGAAGAGGTGGAGGTGGAATCCCTCGTTGGAATTAACGACGCACTGGAAAGTTTAGAACGACAATTGGCTTCT
CTTCAAGATCTCCAACACCAACAGAGATATGAGAGAGAGACCGTCCTGAGTCAGATCGACAGGTCTAGGACCAGTTTGCTT
AACAAGCTCAAGGAGTACAAGGGCGAAGATTGTGAGGCAATACATGAAGCAGCCGCCTTTGCCGGGGAAAAGATCGAGA

ACGACGACGGGCTCATTCTCCCACCGTATAGTGGTCACGTGACAAATTCATTCGTCCTTGACGACCTGTACCCCGCCAACTA<br>CGTTTCCAAACCAAAATGCCTTCACAACGGCTTACGAAGCGATGGTATGACGGAAGACTCAACTCGAACCAACCGTACTCA<br>AAACCGTATCCCTGGGACATCCTCAAGAAACTCTAGTGGGGGAATAAGGTCCTTAATAGGTTGGATGGCTAAAACAGCCGT<br>GATGATTGTAGGTGCCATCTCCATCATGAAGGCAGCAGGATATGAGCCGACTATAGGGAGAAGCGGCATCAAGCTTGACA<br>TAGCAGGGTTACTCGGGAAGGAAGCTGCCGGTGCAAAGGAGCAAGTACCGCCAACACTGCAATGTCCGCCCGGGAAAGT<br>GATGGTGCTTGGGGGGGATGGGCGTGCTCACTGTGTCGTCAAAGAAAGAGTAGAGATACCATTCGGGTCTTCCCTTGACG<br>CTCCAAACGCATCATACGGGTTGGGTgacccagctttcttgtacaaagtggtcccc
>cTPmCherry_CDS_GW_codon_optimized_Nbenth<br>GGGGACAAGTTTGTACAAAAAAGCAGGCTTCACCATGTCGGCCCTCACTACGTCACAACTTGCAACTTCAGCAACTGGATTT<br>GGCATTGCTGACAGGTCGGCGCCCTCCTCGCTCTTGCGTCACGGATTCCAAGGCCTTAAGCCCAGATCACCCGCTGGAGGG<br>GACGCCACGTCCCTTAGCGTTACAACAAGCGCACGTGCTACCCCCAAACAGCAACGTTCAGTTCAAAGGGGGTCTCGTCGG<br>TTTCCGTCTGTGGTTGTTTACGCCACGATGGTTAGCAAGGGTGAGGAAGATAACATGGCCATAATCAAAGAATTCATGCGC<br>TTTAAAGTTCATATGGAAGGTTCCGTCAACGGGCACGAGTTCGAGATCGAGGGCGAAGGAGAAGGAAGACCGTACGAAG<br>GAACGCAGACTGCCAAACTCAAAGTTACGAAAGGCGGGCCGTTGCCTTTTGCGTGGGACATACTTTCTCCTCAGTTTATGTA<br>CGGGTCCAAGGCGTATGTCAAGCACCCGGCAGATATACCTGATTACCTTAAACTCTCGTTTCCTGAAGGTTTCAAATGGGAA<br>AGAGTCATGAATTTTGAGGACGGCGGTGTGGTGACGGTGACCCAAGACTCGTCACTTCAAGACGGAGAGTTCATATACAA<br>AGTGAAACTCCGTGGAACAAATTTCCCTTCAGACGGGCCCGTTATGCAAAAAAAGACTATGGGGTGGGAAGCCTCGTCTGA<br>ACGGATGTACCCCGAGGATGGCGCCTTGAAAGGGGAAATTAAGCAGAGGCTTAAACTCAAGGATGGCGGGCACTACGAC<br>GCGGAGGTGAAGACAACCTATAAGGCCAAGAAGCCAGTTCAGTTGCCCGGAGCTTACAATGTGAACATAAAGCTTGACAT<br>AACTTCCCACAATGAAGACTACACCATTGTTGAACAGTACGAAAGGGCGGAGGGTAGACATTCCACAGGTGGGATGGACG<br>AGCTTTACAAATAAGACCCAGCTTTCTTGTACAAAGTGGTCCCC

Methods S1: Phylogenetic analysis and gene models

PARC6, ARC6, PDV1 and PDV2 protein sequences were retrieved from Ensembl Plants and Phytozome (Goodstein et al., 2012; Yates et al., 2022). Proteins were aligned using ClustalW in MEGA7. Phylogenetic analysis was conducted in MEGA7 (Kumar et al., 2016) using the Maximum Likelihood method based on the JTT matrix-based model (Jones et al 1992). Initial tree(s) for the heuristic search were obtained automatically by applying Neighbour-Join and BioNJ algorithms to a matrix of pairwise distances estimated using a JTT model, and then selecting the topology with superior log likelihood value.

Gene models were taken from Ensembl Plants and domains were annotated using Interpro (Yates et al., 2022; Paysan-Lafosse et al., 2023).

Methods S2: Cloning and construct assembly

To generate the transgenic wheat amyloplast reporter lines, we modified a construct design from Matsushima and Hirano (2019), to use a codon-optimised *mCherry* coding sequence (rather than GFP in the original citation) downstream of the *OsWaxy* transit peptide sequence (sequence in Table S2). This fusion sequence, flanked by attB1 and attB2 recombination sites, was synthesised as a gBlocks fragment (IDT) and recombined into the Gateway entry vector pDONR221 using Gateway BP clonase II (Invitrogen, Thermo Fisher Scientific). The *cTPmCherry* coding sequence was then recombined using Gateway LR clonase II (Invitrogen, Thermo Fisher Scientific) into a modified *pGGG* vector (Hayta et al., 2021), pGGG_AH_Ubi_GW_NosT, encoding for a Hygromycin resistance gene driven by an actin promoter (AH), a gateway cassette for gateway recombination (GW) downstream of the *ZmUbiquitin* promoter (Ubi) and upstream of a Nos terminator (NosT).

TaPARC6-A1, *TaARC6-A1*, *TaPDV1-1-A1*, *TaPDV1-2-A1* and *TaPDV2-A1* sequences were obtained from the RefSeq 1.1 genome from Ensembl Plants (Yates et al., 2022). Codon-optimised coding sequences of *TaARC6-A1*, *TaPDV1-1-A1*. *TaPDV1-2-A1* and *TaPDV2-A1*, flanked by attB1 and attB2 recombination sites, were ordered as a gBlocks fragments (IDT)

(Table S2). These coding sequences were recombined into pDONR221 as above. A codon-optimised sequence of *TaPARC6-A1,* flanked by attL recombination sites and MluI restriction sites (Table S2), was ordered from Genewiz in a pUC-GW-Kan vector. *TaPARC6-A1* and *TaARC6-A1* were recombined into Gateway expression vectors pUBC-YFP, pB7YWG2 and pJCV52; and *TaPDV1-1_A1*, *TaPDV1-2_A1* and *TaPDV2_A1* were recombined into Gateway destination vectors pK7WGF7 and pGWB555 using Gateway LR clonase II. All constructs were confirmed by Sanger sequencing.

Methods S3: Transient transformation of *Nicotiana benthamiana*

Nicotiana benthamiana plants were transiently transformed using *Agrobacterium tumefaciens* (GV3101) carrying the respective constructs. The bacteria were grown at 28°C for 48 h. Cultures were resuspended in MMA buffer (10 mM MES pH 5.6, 10 mM $MgCl_2$, 0.1 mM acetosyringone) at an optical density of 1.0 at 600 nm for confocal microscopy and of 0.3 (0.2 for p19) at 600 nm for protein extraction, and infiltrated into the abaxial side of the leaf using a syringe. Leaves were harvested for confocal microscopy and protein extraction 48-72 h after infiltration.

Methods S4: Gas exchange

Gas exchange measurements were made using an LI-6800P portable photosynthesis system (Li-COR) 40-46 days after germination on the fully expanded flag leaves in the glasshouse (CO_2 concentration ca. 412 ppm, light intensity ca. 280 µmol m^{-2} s^{-1}, temperature ca 21°C) as described in Watson-Lazowski *et al.* (2022). The responses of the CO_2 assimilation rate to step increases in light intensity (AQ) were measured under constant CO_2 conditions (412 ppm). AQ measurements were taken after acclimation of 60-120s at increasing light intensities (0, 20, 50, 75, 100, 150, 200, 500, 750, 1000, 1200, 1500, 1800, 2000 µmol m^{-2} s^{-1}). The response of the CO_2 assimilation rate to step increases of intra-cellular CO_2 (A/Ci) was measured at saturating light (2000 µmol m^{-2} s^{-1}). The A/Ci curves measured at decreasing and increasing CO_2 steps of 400, 300, 200, 100, 50, 0, 400, 400, 600, 800, 1000, 1200 ppm. Maximum rates of carboxylation (Vcmax) and electron transport (Jmax) were calculated from A/Ci curves using the 'Plantecophys' package in R by fitting the raw data to a Farquhar, von Caemmerer, and Berry

photosynthesis model (Farquhar et al., 1980; Duursma, 2015). AQ and A/Ci curves were measured consecutively. Before carrying out A/Ci curves, leaves were allowed to stabilise for 20 min at maximal light intensity (2000 μmol m^{-2} s^{-1}). All measurements were taken 8–14 h after the end of the night.

Methods S5: Starch purification, scanning electron microscopy and polarised light microscopy.

For mature grains, three grains per sample were soaked overnight in double distilled water (ddH_2O) at 4°C, then homogenized in a mortar and pestle with additional ddH_2O. Developing grains were snap frozen in liquid nitrogen at harvest and stored at -80°C. Seeds were thawed immediately before endosperm dissection, and endosperms were homogenized in ddH_2O using a ball mill at 30 Hz for 1.5 minutes. For large amounts of starch, mature grains were first milled into flour (Cyclone Mill Twister, Retsch). Homogenates were filtered through a 100 μm nylon mesh, centrifuged and the pellet was resuspended in 90% (v/v) Percoll, 50 mM Tris-HCl, pH 8. The suspension was centrifuged at 2500 g for 5 min and the pellet was washed twice in 50mM Tris-HCl, pH 6.8, 10 mM ethylenediaminetetraacetic acid (EDTA), 4% sodium dodecyl sulfate (SDS) (v/v), 10 mM dithiothreitol (DTT). The starch pellet was washed and resuspended in ddH_2O.

Granule size distribution was analysed and plotted in relative volume/diameter using the Multisizer 4e Coulter counter (Beckman Coulter). fitted with a 70 μm aperture, operating on either total count mode (measuring a minimum of 500,000 particles) or volumetric mode (measuring a minimum of 1 mL starch suspension). Measurements were conducted with logarithmic bin spacing and were corrected for bin width for presentation on a linear x-axis. A- and B-type granule diameters as well as B-granule contents were extracted by fitting distribution models to the data. Python script available at: https://github.com/DavidSeungLab/Coulter-Counter-Data-Analysis.

The morphology of starch granules was examined by scanning electron microscopy, using a Nova NanoSEM 450 (FEI) scanning electron microscope and the Leica DM6000 microscope for polarised light microscopy. Images were processed using ImageJ software (http://rsbweb.nih.gov/ij/) and Adobe Photoshop 2020.

Methods S6: Total starch quantification, starch composition and amylopectin structure

Grain starch quantification was performed using the Total Starch Assay kit (K-TSTA; Megazyme): Flour (milled in ball mill: 5-10 mg) was suspended in 20 µL 80% ethanol and incubated with 500 µL thermostable α-amylase in 100 mM sodium acetate buffer, pH 5, on a shaking thermomixer at 99 °C and 1400 rpm for 7 min. Amyloglucosidase was added and incubated on a shaking thermomixer at 50 °C and 1000 rpm for 35 min. Samples were centrifuged at 20,800g for 10 min and glucose content was measured in the supernatant using the hexokinase/glucose-6-phosphate dehydrogenase assay (Roche, Basel, Switzerland) to calculate starch content in glucose equivalents.

Amylose content was determined using an iodine-binding method on starch granules dispersed in water, adapted from Washington et al., (2000). Briefly: 1 mg of purified starch (as in S4) was resuspended in 200 µL water, mixed with 200 µL 2 M NaOH solution and incubated at room temperature overnight. The starch slurry was neutralised with 400 µL 1 M HCl. 5 µL of the starch suspension were diluted in 220 µL water and 25 µL Lugol solution (Sigma Life Science). Absorbance was measured at 620 nm and 535 nm and Amylose content was calculated as described in Washington et al., (2000).

Amylopectin chain length distribution was quantified using High Performance Anion Exchange Chromatography with Pulsed Amperometric Detection (HPAEC-PAD) on a Dionex ICS-5000-PAD fitted with a PA-100 column (Thermo). The preparation of debranched samples was carried out as described in Streb et al., (2008).

Methods S7: Analysis of chloroplast morphology

For the analysis of mesophyll chloroplast morphology: separation of mesophyll cells was performed according to Pyke and Leech (1991) with adjustments: Leaf segments of the leaf tip of the 3rd fully developed leaf were harvested into 10% formaldehyde solution (Sigma) in PBS (v/v) and incubated in the dark for 2 h. Formaldehyde solution was replaced by 0.1 M Na_2EDTA, pH 9 and samples were incubated with shaking at 100 rpm and 60 °C for 2 h. Cells were separated by carefully knocking the coverslip during mounting. Mesophyll chloroplasts were imaged using the LSM800 (Zeiss) or the TCS SP8X (Leica) using a 40.0x or 63.0x water immersion objective. Chlorophyll autofluorescence was excited using a white light laser set to 555 nm, 576 nm or 630 nm and emission was detected at 651 nm to 750 nm using a hybrid detector or Airyscan.

Methods S8: Microscopic analysis of amyloplast morphology in developing grain

For the analysis of endosperm amyloplast morphology using confocal microscopy: Developing grain of amyloplast reporter lines (see above) were harvested at 16 DAF, embedded in 4% low melting agarose and sectioned into 150 µm cross sections using the vibratome VT1000s (Leica). Images were acquired immediately after sectioning on the LSM800 using a 63.0 x oil immersion objective (Zeiss). mCherry signal was excited at 561 nm and emission was detected at 562 nm to 623 nm (605 nm).

For analysis of endosperm amyloplast morphology using transmission electron microscopy (TEM), samples were prepared and imaged as described in Chen et al. (2022a):

Developing grain (16 DAF) were harvested into 2.5% glutaraldehyde in 0.05 M sodium cacodylate, pH 7.4. Samples were post-fixed in 1% (w/v) osmium tetroxide (OsO_4) in 0.05 M sodium cacodylate for 2 h at room temperature, dehydrated in ethanol and infiltrated with LR White resin (Agar Scientific, Stansted, UK), using an EM TP embedding machine (Leica, Milton Keynes, UK). LR White blocks were polymerised at 60°C for 16h. For transmission electron microscopy (TEM) ultrathin sections (ca. 90 nm) were cut with a diamond knife and placed onto formvar and carbon coated copper grids (EM Resolutions, Sheffield, UK). The sections were stained using 2% (w/v) uranyl acetate for 1 h and 1% (w/v) lead citrate for 1 min, washed in

water and air dried. Sections were imaged on a Talos 200C TEM (FEI) at 200 kV and a OneView 4K x 4K camera (Gatan, Warrendale, PA, USA).

All images in this manuscript were processed using the ImageJ software (http://rsbweb.nih.gov/ij/) and Adobe Photoshop 2020. Chloroplast images were additionally processed using the Zeiss ZEN software

<u>References</u>

Chen J, Chen Y, Watson-Lazowski A, Hawkins E, Barclay J, Fahy B, Denley-Bowers R, Corbin K, Warren F, Blennow A, Uauy C, Seung D (2022a) The plastidial protein MRC promotes starch granule initiation in wheat leaves but delays B-type granule initiation in the endosperm. BioRxiv. doi.org/10.1101/2022.10.07.511297

Chen J, Watson-Lazowski A, Vickers M, Seung D (2022b) Gene expression profile of the developing endosperm in durum wheat provides insight into starch biosynthesis. BioRxiv. doi.org/10.1101/2022.10.21.513215

Duursma RA (2015) Plantecophys--An R Package for Analysing and Modelling Leaf Gas Exchange Data. PLoS One **10:** e0143346

Farquhar GD, von Caemmerer S, Berry JA (1980) A biochemical model of photosynthetic CO2 assimilation in leaves of C 3 species. Planta **149:** 78-90

Glynn JM, Froehlich JE, Osteryoung KW (2008) Arabidopsis ARC6 coordinates the division machineries of the inner and outer chloroplast membranes through interaction with PDV2 in the intermembrane Space. Plant Cell **20:** 2460-2470

Goodstein DM, Shu S, Howson R, Neupane R, Hayes RD, Fazo J, Mitros T, Dirks W, Hellsten U, Putnam N, Rokhsar DS (2012) Phytozome: a comparative platform for green plant genomics. Nucleic Acids Research **40:** D1178-1186

Hayta S, Smedley MA, Clarke M, Forner M, Harwood WA (2021) An Efficient Agrobacterium-Mediated Transformation Protocol for Hexaploid and Tetraploid Wheat. Current Protocols **1:** 1-15

Jones DT, Taylor WR, Thornton JM (1992) The rapid generation of mutation data matrices from protein sequences. Comput Appl Biosci **8:** 275-282

Kumar S, Stecher G, Tamura K (2016) MEGA7: Molecular Evolutionary Genetics Analysis Version 7.0 for bigger datasets. Molecular biology and evolution **33:** 1870-1874

Matsushima R, Hisano H (2019) Imaging Amyloplasts in the Developing Endosperm of Barley and Rice. Scientific Reports **9:** 3745

Paysan-Lafosse et al. (2023) InterPro in 2022. Nucleic Acids Res **51:** D418-D427

Pyke KA, Leech RM (1991) Rapid Image Analysis Screening Procedure for Identifying Chloroplast Number Mutants in Mesophyll Cells of Arabidopsis thaliana (L.) Heynh. Plant Physiol **96:** 1193-1195

Streb S, Delatte T, Umhang M, Eicke S, Schorderet M, Reinhardt D, Zeeman SC (2008) Starch granule biosynthesis in Arabidopsis is abolished by removal of all debranching enzymes but restored by the subsequent removal of an endoamylase. Plant Cell **20:** 3448-3466

Washington JM, Box A, Karakousis A, Barr AR (2000) Developing Waxy Barley Cultivars for Food, Feed and Malt. Barley Genetics VIII**:** 303-306

Watson-Lazowski A, Raven E, Feike D, Hill L, Barclay JE, Smith AM, Seung D (2022) Loss of PROTEIN TARGETING TO STARCH 2 has variable effects on starch synthesis across organs and species. J Exp Bot **73:** 6367-6379

Yates at al. (2022) Ensembl Genomes 2022: an expanding genome resource for non-vertebrates. Nucleic Acids Res **50:** D996-D1003
